# Editorial: Exposure, Risks, and Drivers of the Mobile Antimicrobial Resistance Genes in the Environment—a Global Perspective

**DOI:** 10.3389/fmicb.2021.803282

**Published:** 2021-11-30

**Authors:** John P. Brooks, Lisa M. Durso, Abasiofiok Mark Ibekwe

**Affiliations:** Agricultural Research Service, United States Department of Agriculture, Washington, DC, United States

**Keywords:** AMR, environment—agriculture, pathogen, indicator, manure, water, indicator, crop

*The life and work of Heather K. Allen*

*October 24, 1980 – March 7* ^*th*^ *, 2020*

In 2010, Dr. Heather K. Allen published a foundational review paper on the emerging field of environmental antibiotic resistance. “Call of the wild: antibiotic resistance genes in natural environments” (Allen et al., 2010) introduced a conceptual framework for understanding and assessing antibiotic resistance in water, soil, air, and wildlife, at a time when the focus of antibiotic resistance work was primarily on human clinical settings. The paper provided a framework that built on Dr. Allen's Ph.D. work with Dr. Jo Handelsman, integrating microbial ecology with the then-new metagenomic sequencing tools to probe antibiotic resistance in natural and agricultural settings. The paper defined priority Research Topics that have guided a generation of researchers in the field and has received over 1,900 citations to date. Dr. Allen's “Call of the Wild” highlighted the important role of naturally occurring antibiotic resistance; the diversity and role of antibiotic resistance genes in bacterial communities from both naturally occurring and human-impacted sites; and the need to better understand factors contributing to the spread of antibiotic resistant bacteria and their genes in the environment. These are the topics covered by this current Frontiers Research Topic “*Exposure, Risks, and Drivers of the Mobile Antimicrobial Resistance Genes in the Environment—a Global Perspective*.”

After leaving the Handelsman laboratory, Dr. Allen took a position at the USDA-Agricultural Research Service in Ames Iowa. While the focus of her antibiotic resistance research shifted to the gut microbiome of swine, she remained a thought leader in the field of environmental antibiotic resistance. During her short career she received numerous awards for her work on antibiotic resistance, including the prestigious Arthur S. Flemming award for scientific achievements and the Presidential Early Career Award for Scientists and Engineers.

Heather had a sharply analytical mind, visionary creativity, and a talent for writing that changed the conversation around environmental antibiotic resistance. She leaves a lasting and positive influence on the field, both through her innovative research, and through her gracious collegiality.

The potential sources and movement of antimicrobial resistant (AMR) bacteria and antibiotic resistance determinants (ARD) through the soil and water can lead to a seemingly endless web of interconnected public health consequences and the environment. While we, generally, are aware of the many sources of AMR, we still don't understand the environmental fate and ultimately the public health consequences of AMR bacteria. The goal of this Research Topic is to present current research on the source of AMR from both anthropogenic and animal sources, elaborate on the potential movement through the environment, and present new approaches to surveying for AMR and ARD.

## Animal and Human Sources of AMR

The human and animal sources of AMR have long been tied to spread to the environment. Long considered a major source for environmental pollution, these respective sources are key control points in the reduction of AMR in the environment. Animal feeding operations and municipal wastewater treatment plants are considered “ideal” milieu for promoting the proliferation of AMR and selection and exchange of ARD. In the current special section, we have gathered international contributions from teams working on the presence of AMR in the environment, with those focusing on farm and wastewater treatment plants. While foodborne bacterial pathogens are typically investigated, and rightly so, we have papers focused on commensal bacteria as well. We have gathered papers covering the diversity of gene cassettes in commensal *E. coli* at swine farms (Zhang et al.) to whole genome sequencing of common foodborne pathogen indicator species, such as vancomycin resistant *Enterococcus* spp. (Foka et al.). Poultry production in Brazil is covered by original research demonstrating extended spectrum beta lactamase producing *E. coli* in samples through production and the immediate food production environment (Gazal et al.). On farm anaerobic treatment of manure and effects of wastewater treatment plants on ARD and their surveillance were investigated by Agga et al.; Majeed et al., respectively. Surveillance of ARD and AMR bacteria utilizing metagenomics is a highly sought-after topic, particularly while trying to understand the context of baseline levels of these pollutants in the environment. Finally, we have near retail meat products destined for human consumption and potential for AMR exposure (Wang et al.), and review of the current literature summarizing antibiotic resistance genes in animal manure and potential fate following land application.

## AMR in Water

One primary interface between the public and AMR bacteria is in watersheds and surface freshwater. Manure and wastewater treatment by products are often disposed of in pasture and row crop fields which are buffered by surface water bodies. One process that drives AMR bacteria and ARGs from manure is via rain associated runoff. In the current special section, we have an article covering the effect of long-term pasture management on ARD in runoff (Yang et al.). We have also gathered similar research in an urban watershed impacted by wastewater treatment plant recharge (Mukherjee et al.). These two studies focused on the targeting of ARD in the environment. A method to control surface runoff is irrigation return flows, however return flows are known to harbor high levels of contaminants and bacteria. A study by Dungan and Bjorneberg investigated *E. coli* and enterococcal AMR profiles in return flows.

## New Tools for Understanding Dissemination of Antibiotics and Antibiotic Resistance from the Broad Environment to Crops

More advanced knowledge in chemistry, molecular microbiology, metagenomics, and bioinformatics are needed to understand the ecology of transmission of antibiotics and ARGs from the environment to crops and potentially to human. Matrices such as manures, biosolids, and wastewater are highly significant sources of antibiotics and ARDs. In addition to these, direct gene transfers between microbes present in the waste material and the soil can also develop resistance owing to the selective pressure applied by the presence of antibiotic compounds in the waste material (Duan et al., 2017). The occurrence of antibiotics in such systems may lead to genetic changes in sensitive bacteria (Martinez, 2009). MGEs would further enhance the dissemination and promotion of genetic recombination of ARGs via horizontal gene transfer (HGT) (Vikesland et al., 2017). In a minireview, Bartkova et al. outlined the current technologies used to characterize microplastics based ecosystems termed “plastisphere” and their AMR promoting elements and highlighted emerging technologies that could be useful for systems-level investigations of AMR in the plastisphere. When analyzing these ecosystems scientists can integrate metagenomics and metatranscriptomics with machine-learning tools such as DeepARG or any other artificial intelligent tools (AI) to find the existing and novel ARGs and MGEs for future AMR work (Arango-Argoty et al., 2016; Cuadrat et al., 2020).

In the broad environment, livestock manure application to cropland for soil fertility presents a concern that ARG and bacteria may proliferate and be transported in the environment (Miller et al.). For instance, swine manure application to agricultural soil can introduce diverse set of *tet* resistances genes into low *tet* resistance agricultural soil. One of the major concerns after manure or wastewater application to agricultural soil is the dissemination of chemicals of emerging concern (CEC) through the food chain. This was observed when 11 fosfomycin-resistant *Citrobacter freundii* strains from 270 samples were identified from flowers and the retail environment (Cheng et al.). The authors noted that these isolates were multidrug-resistant, and most were simultaneously resistant to fosfomycin, cefotaxime, ciprofloxacin, and amikacin, therefore potentially posing a public health threat to workers. It is interesting to note that there is a high prevalence of widespread acquired resistance genes among bacteria such as *Enterococcus* strains exposed to anthropogenic antibiotic pressure in the environment (Aun et al.). However, they noted that *E. faecium* strains in their dataset were found within the same host species or environmental origin, confirming the previous findings that *E. faecalis* from the same ST can be found in human as well as in other animal species, while *E. faecium* strains tend to be host specific (Hammerum, 2012). The biggest concern of AMR is their transferability from the environment; one of the routes may be through fresh produce that are eaten raw or with minimally processed. Due to high water demands especially in the southwestern part of the United States, there is a high demand for new water reuse programs, including treated municipal wastewater usage. The use of wastewater to grow leafy greens could pose potential health risks due to the high probability of AMR in such wastewater (Summerlin et al.). These authors suggested that successful reuse of wastewater in agriculture will depend on appropriate mitigation and management strategies to guarantee safe water supply. In an integrated study to understand factors influencing the occurrence, distribution, and fate of antibiotic resistance genes in vegetable production systems, Wind et al., suggested that pre-harvest and potentially post-harvest interventions may be warranted to minimize risk of propagating antibiotic resistance in the food chain. They demonstrated a holistic approach as the best options to identifying key control points for the propagation of ARGs in vegetable production systems, identifying potential ARG-MGE combinations that could inform future surveillance. They suggested that agroecosystems are a key reservoir of antibiotic resistance and appropriate mitigation strategies are needed to limit potential for ARGs to spread and negatively influence human and animal health.

All told, this special section covers a wide swath of research from AMR and ARD in wastes and products derived from both human and animal sources, and their potential movement through the environment, ultimately culminating in potential public health consequences.

## Author Contributions

JB, LD, and AI were involved in the conception, execution, and editing. All authors contributed to the article and approved the submitted version.

## Conflict of Interest

The authors declare that the research was conducted in the absence of any commercial or financial relationships that could be construed as a potential conflict of interest.

## Publisher's Note

All claims expressed in this article are solely those of the authors and do not necessarily represent those of their affiliated organizations, or those of the publisher, the editors and the reviewers. Any product that may be evaluated in this article, or claim that may be made by its manufacturer, is not guaranteed or endorsed by the publisher.

## References

[B1] Allen H. K. Donato J. Wang H. H. Cloud-Hansen K. A. Davies J. Handelsman J. (2010). Call of the wild: antibiotic resistance genes in natural environments. Nat. Rev. Microbiol. 8, 251–259. 10.1038/nrmicro2312 20190823

[B2] Arango-Argoty G. Singh G. Heath L. S. Pruden A. Ziao W. Zhang L. (2016). MetaStorm: a public resource for customizable metagenomics annotation. PLoS ONE 11:e0162442. 10.1371/journal.pone.0162442 27632579 PMC5025195

[B3] Cuadrat R. R. C. Sorokina M. Andrade B. G. (2020). Supporting data for Global ocean resistome revealed: exploring antibiotic resistance gene abundance and distribution in TARA Oceans samples. GigaScience Database 2020:12. 10.5524/100739 PMC7213576 32391909

[B4] Duan M. Li H. Gu J. Tuo X. Wang X. (2017). Effects of biochar on reducing the abundance of oxytetracycline, antibiotic resistance genes, and human pathogenic bacteria in soil and lettuce. Environ. Pollut. 224, 787–795. 10.1016/j.envpol.2017.01.021 28284554

[B5] Hammerum A. M. (2012). Enterococci of animal origin and their significance for public health. Clin. Microbiol. Infect. 18, 619–625. 10.1111/j.1469-0691.2012.03829.x 22487203

[B6] Martinez J. L. (2009). Environmental pollution by antibiotics and by antibiotic resistance determinants. *Environ*. Pollut. 157, 2893–2902. 10.1016/j.envpol.2009.05.051 19560847

[B7] Vikesland P. J. Pruden A. Alvarez P. J. J. Aga D. Bürgmann H. Li X. D.. (2017). Toward a comprehensive strategy to mitigate dissemination of environmental sources of antibiotic resistance. Environ. Sci. Technol. 51, 13061–13069. 10.1021/acs.est.7b03623 28976743

